# Advancing Digital Access to Physical Therapy via Virtual and Extended Reality Technology: Prototype Development and Usability Evaluation

**DOI:** 10.2196/73783

**Published:** 2025-12-05

**Authors:** Victoria Lynn Tiase, Julie M Fritz, Jesse Ferraro, Gregory Bayles, Ahmad Alsaleem, Guilherme Del Fiol, Kensaku Kawamoto, Darrel Brodke, Brook Martin, Roger Altizer

**Affiliations:** 1 Department of Biomedical Informatics, School of Medicine, University of Utah, 421 Wakara Way, Salt Lake City, UT, 84112, United States, 1 801-585-3945; 2 Physical Therapy and Athletic Training, College of Health, University of Utah, Salt Lake City, UT, United States; 3 Population Health Sciences, School of Medicine, University of Utah, Salt Lake City, UT, United States; 4 Division of Games, College of Architecture and Planning, University of Utah, Salt Lake City, UT, United States; 5 School of Computing, College of Engineering, University of Utah, Salt Lake City, UT, United States; 6 Department of Orthopaedics, School of Medicine, University of Utah, Salt Lake City, UT, United States

**Keywords:** virtual reality, physical therapy, low back pain, user-centered design, access to care

## Abstract

**Background:**

The United States faces significant challenges in physical therapy (PT) access due to high demand, a shortage of professionals, and patient-related obstacles, which can adversely affect recovery and function. Limited access to PT may lead to increased dependence on medications for pain management, highlighting the need for nonpharmacologic options to reduce opioid overprescribing. Low back pain, a leading cause of disability and high medical costs, is a common reason for requiring PT following surgery. Studies have shown that virtual reality (VR)–guided movements can improve motor function and reduce pain intensity.

**Objective:**

The objective of this study was to design, develop, and evaluate a VR-based prototype for individualized postoperative PT for patients recovering from back surgery to investigate its potential to improve convenience, access, and health outcomes in future research.

**Methods:**

Study methods involved participatory design and development of VR software for PT back exercises using the design box method, an inductive, problem-oriented collaborative design approach. A usability evaluation of the resulting prototype was conducted with patients recovering from back surgery using a think-aloud protocol and usability survey.

**Results:**

Six participants evaluated the VR prototype and reported usability challenges that included mismatched VR boundaries, limited familiarity with VR, and difficulties with the headset and hand controls. The System Usability Scale resulted in a total usability score of 58.3 out of 100, indicating a below-average score (68 being average).

**Conclusions:**

In the design and evaluation of a VR-based PT prototype, we found that while participants were enthusiastic, they faced usability challenges due to insufficient instructions and difficulties operating the VR device, highlighting the need for effective onboarding and extensive prototype testing to improve accessibility and engagement in health care. Future evaluations will investigate disparities among different groups to ensure accessibility and effectiveness for all users.

## Introduction

The United States is currently experiencing significant challenges in physical therapy (PT) access due to high demand; a shortage of trained professionals; and obstacles related to patients’ location, scheduling, and mobility issues [1,2]. Many health systems across the country struggle to provide the recommended PT care, leading to longer wait times and extended periods without care, which can adversely affect patients’ recovery and function [3,4]. This issue is particularly concerning for older adults who may rely on PT to maintain their independence [5,6]. As PT is an evidence-based, nonpharmacological option for pain management, limited access may drive patients to increasingly depend on medications or other pain care options [7]. Expanding access to nonpharmacologic options such as PT is a crucial aspect of reducing overprescribing of opioids and other pain medications and reducing the impact of pain on the quality of life of individuals [8-10]. Despite efforts to train more physical therapists, effective strategies that leverage scalable technology, such as extended and virtual reality (VR), are still needed to overcome all the barriers that limit access to PT services [2].

Extended reality, an umbrella term that encompasses all immersive technologies, including augmented reality and VR technology, is rapidly transforming health care in numerous innovative ways [11]. VR, a digital environment that replaces the physical world, is being used to treat phobias and mental health disorders [12]. Additionally, VR facilitates health behaviors by encouraging physical activity within enjoyable, engaging virtual environments [13]. Since 2019, the adoption of VR in health care has grown, for example, with the emergence of VR-based health care providers who interact with patients virtually, eliminating the need for physical presence [12]. VR has shown promising results in orthopedic rehabilitation for chronic neck pain and shoulder impingement syndrome and is considered a valuable tool for upper-extremity neurorehabilitation and pain distraction [13-15]. With the right equipment and training, VR offers a novel and patient-centric solution to expand access to PT care beyond what basic PT health applications can offer.

Low back pain is one of the most common reasons why people seek PT as it is the leading cause of disability and one of the costliest medical conditions, with Medicare data revealing that 94% of patients with a new diagnosis of back pain have an outpatient PT episode within a year [12,16,17]. With this high need for PT, low back pain is a plausible condition to explore the use of VR visits. Recently, multiple studies have demonstrated that VR movements can improve motor function outcomes, significantly reducing pain intensity in patients with low back pain [17-19]. Additionally, there is evidence suggesting that remote PT via telehealth is comparable to in-person PT, with patients showing some willingness to use this approach [20-22]. Developing a VR-based PT program for patients with chronic low back pain could be an effective strategy to expand access to PT care to manage this condition.

In this study, ADAPT XR (Advancing Digital Access to Physical Therapy via Virtual and Extended Reality Technology), we designed and developed a VR-based prototype to provide customized postoperative PT for patients recovering from back surgery. This study consisted of two stages: (1) participatory design and development of VR software for PT back exercises and (2) usability evaluation of a prototype VR-based PT intervention with patients recovering from back surgery. We hypothesized that the VR-based care approach for postsurgical back rehabilitation would be well received by patients.

## Methods

### Participatory Design and Development

In the first phase, we used a participatory design approach that was user-centric, visual, iterative, and collaborative, fostering end-user engagement and design thinking [23]. We conducted 2 focus group sessions using the design box method, a technique originally developed for video game design that facilitates end-user collaboration in prototype design and development [24,25]. Given the gaming aspects of VR, we felt that the design box methodology would be best suited for this application. One focus group comprised physical therapists, and the second consisted of individuals who had previously used PT services following back surgery.

**Figure 1.**
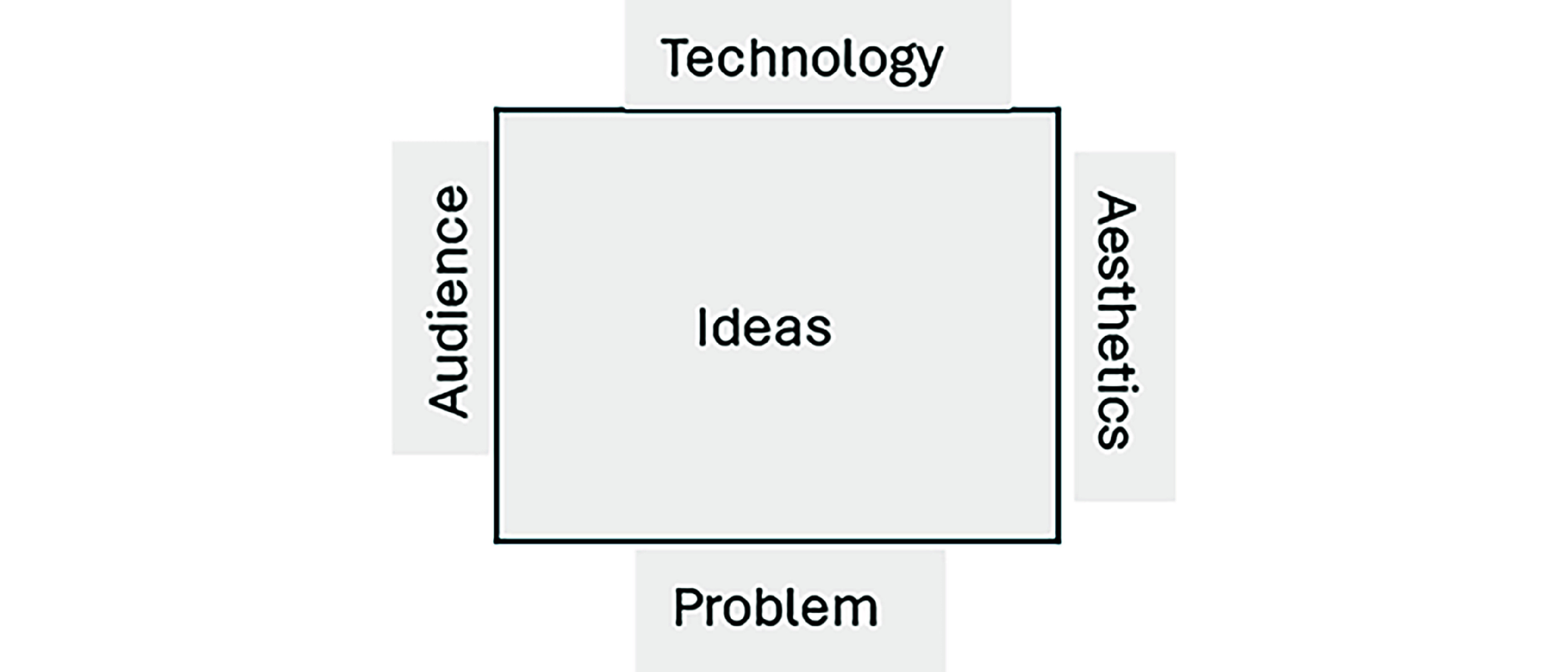
Design box methodology with 4 walls or constraints.

The design box method, created by Altizer et al [25] at the University of Utah, advances creative ideation by allowing groups to generate ideas iteratively and design prototype features using a visual tool. It is an inductive design methodology built around the notion that good design solves a problem. This method includes drawing 4 “walls” or constraints on an electronic whiteboard: problem, technology, audience, and aesthetics (Figure 1). The moderator, a member of the research team, gathers input from participants for each wall in sequence, recording their contributions in real time for everyone to see. Once theoretical saturation is reached and no new ideas are generated, design box focus group participants refine the ideas to arrive at a clear design that the entire group supports, otherwise referred to as the “pitch.” By leveraging the 4 walls as constraints, participants are free to pitch any idea that addresses the problem statement, technical needs, audience needs, and agreed-upon aesthetics.

For the physical therapist focus group, we recruited therapists with outpatient experience in providing PT. Using purposive sampling, we targeted physical therapists at the University of Utah in Salt Lake City, Utah, through email and word of mouth. Additionally, we used a snowball sampling technique, asking existing study subjects and local contacts to identify other potential participants. The inclusion criteria were individuals who were aged ≥18 years, English speaking, and licensed to practice PT.

For the second focus group, we recruited individuals who had undergone PT following surgery for back pain. Using both purposive and snowball sampling, we asked health care providers to refer patients who met the criteria. Other inclusion criteria were individuals who were aged ≥18 years, English speaking, and able to meet via Zoom (Zoom Video Communications).

Once the design box focus group sessions were completed, prototype development for the VR application commenced. To facilitate development, the notes and whiteboards from the design box sessions were summarized and analyzed to determine which ideas or features were common and fit within the scope of the project. The analysis of design boxes shares similarities with inductive approaches as the core design of the project emerges from commonalities in and observations of the design box sessions [26]. The core design is broken down into features, which in turn are put into a production backlog, facilitating agile software development [27].

A team of 3 graduate students, 1 producer, and 1 engineer from the Therapeutic Games and Apps Lab at the University of Utah reviewed and aligned the design box results with the capabilities of the Meta Quest VR device (Reality Labs), adding further nuance to the design and backlog. The Meta Quest VR device, a commercial off-the-shelf technology, was selected due to its market prominence, making it easy for translation and adoption. It should be noted that members of the research team were also members of the development team, allowing for negotiation between features that participants in the design box sessions wanted and what was technically feasible and within the project’s scope.

To decide which PT exercise to prototype, the team met with physical therapists to review which exercises were used in postsurgical back pain PT and could be tracked using the controllers and headset of the Meta Quest VR system. The development team at the Therapeutic Games and Apps Lab created a detailed design plan for the leg squat exercise, identified by the PT experts as commonly used in postsurgical back pain PT. The plan was based on 2-week sprint cycles of software development, a key aspect of agile development [27]. Sprint cycles allow for work to be broken down into scheduled iterations, which in turn allow for rapid development and the flexibility to modify any features in between sprint cycles based on user, expert, and team feedback [27]. A total of 5 sprint cycles were executed for this project. Using the development platform Unity (Unity Technologies), the team programmed the prototype’s functionality, integrating VR-specific features such as hand tracking and 6-df movement. Rigorous testing was conducted to ensure smooth performance and a safe, immersive experience, addressing any technical flaws or issues. A functioning prototype for the Meta Quest device was completed and used during the usability evaluation phase.

### Usability Evaluation

Following the development of the prototype, participants who had previously undergone PT following surgery for back pain were recruited for a 1-hour in-person session to take part in a usability evaluation. Recruitment strategies included soliciting patients from the University of Utah Orthopedic Center and the researchers’ professional contacts. Using both purposive and snowball sampling, we asked health care providers to refer patients who met the criteria. Inclusion criteria specified individuals aged ≥18 years who were English speaking and had completed PT for postsurgical back pain. Patients currently undergoing treatment for postsurgical back pain were excluded from the study so as to not interfere with treatment regimens.

The usability evaluation comprised several steps, including an interview using a think-aloud protocol and a postuse survey [28,29]. To ensure adherence to study procedures, the research team developed guidance documents for the Meta Quest setup and cleaning between uses, think-aloud protocol questions, postuse survey questions, and safety procedures. Three members of the research team, all trained in usability methods, were present for each of the usability evaluation sessions.

Once eligibility was confirmed and consent was completed, participants were introduced to the general use of the Meta Quest headset and hand controllers. Participants were asked to perform the tasks as instructed by the VR prototype. Subsequently, participants were asked a series of questions and probes in conjunction with the think-aloud protocol to facilitate a deeper exploration of their interaction with the tool. Examples of probes included “What do you think that means?” or “Is that what you expected to see?” The questions were designed to progress from general to more specific information.

Additionally, each participant completed a 10-item validated survey tool, the industry-standard System Usability Scale (SUS), to assess factors such as perceived user performance, perceived decision quality, and perceived task completion time [30]. Responses were collected using REDCap (Research Electronic Data Capture; Vanderbilt University), a secure web application [31-33]. Given the formative and diagnostic nature of this study, our primary focus was on identifying significant usability issues. Following SUS guidelines, we calculated a percentile ranking and average score for the responses.

Conceptually, we used thematic saturation, ceasing recruitment when no new insights or themes emerged from the data [34]. Unlike quantitative studies, qualitative studies typically have smaller sample sizes, and guidance for qualitative sample sizes indicates that above 5 is considered adequate for identifying a large portion of usability issues [35-37]. At the conclusion of the usability evaluation, we advised participants to wait for 30 minutes before attempting to drive home or operate heavy machinery as disorientation or nausea could pose safety risks after using a VR device.

Verbatim transcripts were anonymized and verified by a second investigator. Free-text segments were excerpted from the transcripts for content analysis. Using a securely stored spreadsheet, 3 members of the research team coded the transcripts based on the 9 concepts of the Health Information Technology Usability Evaluation Model [38,39]. To enhance rigor, coders first underwent a group coding session to establish a shared understanding of the coding framework. Instead of calculating intercoder reliability, we conducted iterative consensus coding. Through group consensus, we labeled segments as “positive,” “negative,” and “neutral” for the 9 concepts. Regular meetings with the coders were held to discuss and resolve discrepancies, refine the codebook, and ensure consistent application of the conceptual categories in line with qualitative best practices.

### Ethical Considerations

This study received ethics approval from the University of Utah Institutional Review Board (00165806). After the usability evaluation, participants were emailed a US $50 gift card as compensation for taking part. All participants provided written informed consent prior to participation and were informed of the study purpose, procedures, potential risks, and their right to withdraw at any time without penalty. To protect participant confidentiality, all raw data were de-identified prior to analysis. A separate linkage file connecting participants to study IDs was stored in a secure, encrypted location accessible only to the study team. No identifiable information was shared outside the research team.

## Results

### Participatory Design and Development

Seven individuals met the inclusion criteria for the design box focus groups: 3 (43%) physical therapists and 4 (57%) individuals who had undergone PT for postsurgical back pain. Each of the sessions lasted approximately 90 minutes and was audio recorded. The sessions were conducted using Zoom, and the moderator used Miro as the electronic whiteboard. Idea refinement for each of the constraints is shown in Table 1. All participants enthusiastically supported the vision of designing VR-enabled PT exercises.

**Table 1.** ADAPT XR (Advancing Digital Access to Physical Therapy via Virtual and Extended Reality Technology) design box constraints and ideas resulting from the focus group sessions.

Constraint	Final idea convergence
Problem	Fear of reinjury if unsupervised
Technology	Novelty; simple to use
Audience	Convenient; fit into schedule
Aesthetics	Fun; mini games in between repetitions to keep the intervention engaging

**Figure 2.**
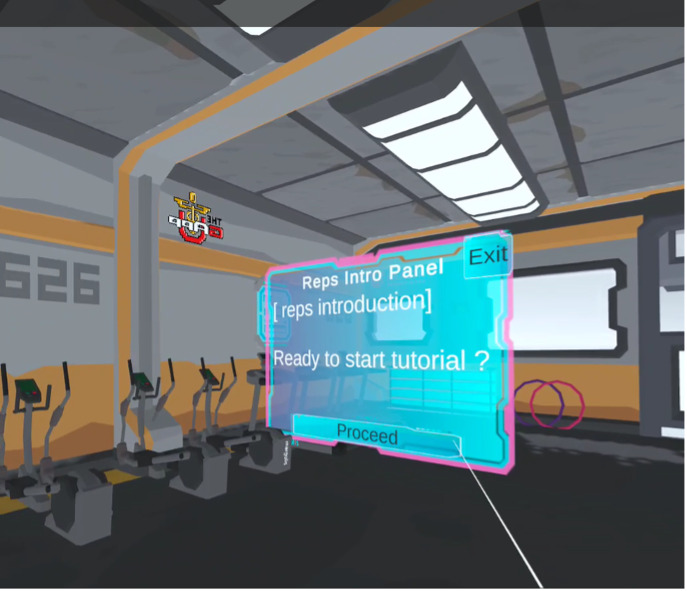
ADAPT XR (Advancing Digital Access to Physical Therapy via Virtual and Extended Reality Technology) virtual reality prototype menu.

**Figure 3.**
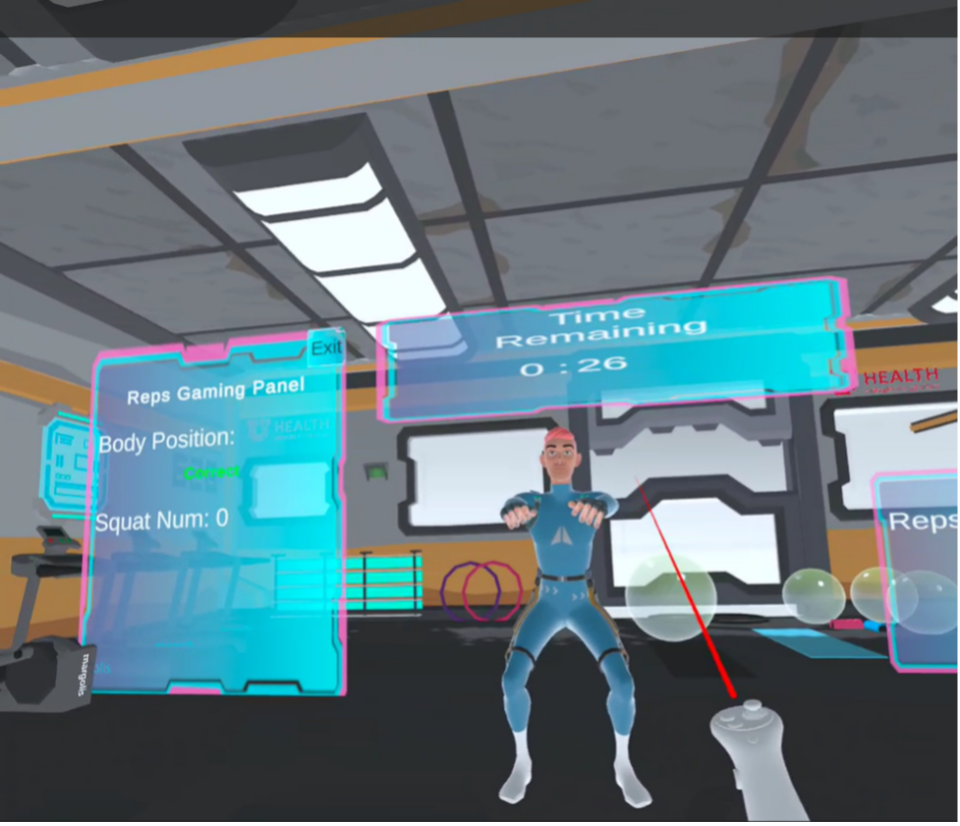
ADAPT XR (Advancing Digital Access to Physical Therapy via Virtual and Extended Reality Technology) avatar and exercise demonstration.

The pitches from the design box session were reflected in the final prototype design, functionality, and user experience. The VR prototype contained basic menus, buttons, and other interactive elements (Figure 2). The virtual environment resembled a high-technology gym setting with a futuristic avatar used to demonstrate the leg squat exercise (Figure 3). This aesthetic was chosen based on feedback from the design box participants. Participants wanted the environment to be more “fun and exciting” than a standard gym. Additionally, participants mentioned that while some had positive associations with PT gyms, others had negative ones due to postsurgical discomfort.

The VR software leveraged visual and auditory feedback to indicate users’ actions in the software. Buttons would animate to appear pressed, and audio was used to give additional feedback. The users were able to view the avatar (or coach) in front of them demonstrating the exercise and could mirror their movements. Users had the ability to touch buttons or use a virtual laser pointer to click on user interface elements that were out of reach. The VR software enabled users, who may be experiencing physical discomfort, to interact with the user interface without needing to stretch or bend. The experience began with an introduction to the environment and a demonstration of how to perform a leg squat, along with advice on how to do so properly and safely. The users were then able to practice along with the avatar in front of them. Users could decide between 2 modes for their exercise: a timed mode and a counter mode. This choice allowed users to perform as many squats as they wanted to in a given period or take their time and do a number of repetitions without a timer. The system tracked their movements and automatically counted each squat.

### Usability Evaluation

Six individuals were recruited for the in-person usability evaluation sessions with 30 to 45 minutes of VR exposure at the orthopedic clinic. Participants were mostly female (n=5, 83%) and aged >50 years (n=4, 67%; mean age 58.3, SD 11.7 y), and 100% (6/6) identified as non-Hispanic White. Overall, participants were enthusiastic about using the VR tool, noting that it was “fun” and “makes exercise less boring.” However, participants reported usability issues related to information needs, especially a lack of clarity on where to move, what to do next, and how many repetitions were completed. Usability issues were also identified concerning the use of the hand controllers and wearing the headset. Representative quotes for each of the 9 Health Information Technology Usability Evaluation Model concepts, along with their descriptions, are shown in Table 2.

The SUS scores were analyzed by averaging the responses from the 6 participants by question (Table 3) and calculating a total adjusted score. Using the Nielsen Norman Group usability guidelines [40], we found a total usability score of 58.3 for the VR prototype, indicating a below-average score [41].

**Table 2.** Health Information Technology Usability Evaluation Model concepts, descriptions, and representative quotes resulting from the ADAPT-XR (Advancing Digital Access to Physical Therapy via Virtual and Extended Reality Technology) virtual reality (VR) tool usability evaluation.

Concept	Description	Representative quote
Error prevention	The VR tool offers error management; error correction; or error prevention, such as instructions or reminders, to assist users performing tasks.	“Yes, it says to move to the area and then lights up a few seconds afterwards. It says move to highlighted area to continue and I’m right on it.” [Neutral]
Completeness	The VR tool is able to assist users in successful completion of tasks.	“Congratulations, now it says I’m done, I didn’t even get to do any squats.” [Negative]
Memorability	Users can remember how to perform tasks in the VR tool easily.	“Okay I’m squatting this is about as far as I can go. Okay, I’m just going to keep going with him. He had a good suggestion so I like that oh I get it so he wants me to keep going.” [Positive]
Information needs	The information offered by the VR tool is for basic task performance or to improve task performance.	“He is informative when he walked me through how to do, like it was helpful how he told me to keep my feet down, tuck in my hips so that was helpful.” [Positive]
Flexibility and customizability	The VR tool provides more than one way to accomplish tasks, which allows users to operate the system as preferred.	“The instructions probably go a little fast because it’s reading the text and I tend to read the text and not listen to the guy as much, and so the avatar was in front of the text and that was annoying.” [Negative]
Learnability	Users are able to easily learn how to operate the VR tool.	“Okay this is the first problem I see a B but it really looks like it should be X so it’s not as it’s written left is X and right is Y and, on the thing, it shows X is below Y.” [Negative]
Performance speed	Users are able to use the VR tool efficiently.	“While it was doing the tutorial, the avatar was showing you how to do it at the same time and then it went into a squat and then it gives you more instructions and you have to hold the squat for a long time. Like I was thinking…. Whoa….” [Negative]
Competency	Users are confident in their ability to perform tasks using the VR tool.	“Oh wow off balance, I don’t think I can do that.” [Negative]
Other outcomes	Other VR tool-specific outcomes such as user expectations.	“This would be good for patients out of state.” [Positive]

**Table 3.** Means and SDs of System Usability Scale (SUS) responses (5-point Likert scale: 1=“strongly disagree,” 2=“disagree,” 3=“neither agree nor disagree,” 4=“agree,” and 5=“strongly agree”) for the ADAPT-XR (Advancing Digital Access to Physical Therapy via Virtual and Extended Reality Technology) virtual reality (VR) tool usability evaluation.

SUS item	Score, mean (SD)
“I think that I would like to use this VR tool frequently.”	3.83 (1.07)
“I found the VR tool unnecessarily complex.”	3.00 (1.00)
“I thought the VR tool was easy to use.”	3.17 (0.90)
“I think that I would need the support of a technical person to be able to use this VR tool.”	3.00 (1.15)
“I found the various functions of this VR tool were well integrated.”	4.17 (0.37)
“I thought there was too much inconsistency in this VR tool.”	2.67 (1.11)
“I would imagine that most people would learn to use this VR tool very quickly.”	3.33 (1.37)
“I found the VR tool very cumbersome to use.”	3.00 (1.15)
“I felt very confident using the VR tool.”	3.17 (1.07)
“I needed to learn a lot of things before I could get going with this VR tool.”	2.67 (1.11)

## Discussion

### Principal Findings

In this study, we identified several challenges while evaluating the usability of a prototype version of the ADAPT XR VR tool. Although some participants gave positive feedback, we found usability issues related to operating the device and navigating the physical space. As the VR environment requires boundaries to enable user movement while maintaining immersion, settings must be applied in advance of use. However, the boundaries in the VR prototype did not match the physical space available for the evaluation sessions. Additionally, the boundaries were overly restrictive, causing participant discomfort and frustration with where to move. Future iterations need to address these boundary issues to ensure accurate usability results as well as participant safety.

Usability was also impacted by participants’ overall familiarity with VR. They found the headset difficult to adjust and uncomfortable to wear. Additionally, there were challenges in using the hand controls and selecting menu options. This was reflected in the SUS scores, which indicated the complexity of the VR tool and the need for significant learning before use. Evaluating the usability of VR tools can be complex given the multiple hardware and software components. However, our usability score of 58.3 is in line with other technology prototype scores, and we believe that it provides an adequate baseline to improve upon [42]. Future evaluations would benefit from offering a general orientation to VR hardware and environments before testing a particular application.

### Comparison to Prior Work

To enhance usability during the development process, we used design box participatory methods to investigate user expectations alongside a usability evaluation of the initial prototype. While the design and development of the exercises were feasible, the usability aspects required more attention than anticipated. As found in other studies, human factors issues, such as poor interfaces, lack of user feedback, and physical limitations, are significant barriers to acceptability in patient-facing technologies and can negatively impact health outcomes [43]. Additionally, ergonomic issues have been cited in VR applications for office work and teaching [44,45]. Canniff and Cliburn [44] reported discomfort with the headset as the most important finding of their evaluation of the Oculus Quest with students. To our knowledge, this is the first study to report similar issues in a health care context. Although we acknowledge that some of the usability issues are limitations of the current VR technology available, in future testing, we intend to place a focus on the physical aspects of the tool to understand the impact of comfort on continued use.

### Future Directions

As this study evaluated our initial prototype design, we plan to conduct multiple rounds of prototype testing as we iteratively refine the tool, and we expect usability to improve with further rounds of refinement. We will focus on evaluating a single exercise, the leg squat, within the VR tool to gain detailed insights into its usability by incorporating feedback mechanisms to assess whether the exercise was done correctly and the individual’s ability to balance. This targeted approach will allow us to make specific adjustments and better understand user experiences. Additionally, we will implement a comprehensive onboarding process to familiarize users with VR devices and environments before evaluating the specific application. We believe that onboarding will be critical for VR tool usability until VR devices become more intuitive and are widely adopted. We plan to further investigate how the use of VR tools may exacerbate disparities among different groups, such as technology-savvy individuals, rural communities, and those with a lower socioeconomic status. This will help us identify and mitigate potential barriers to access and usability. We are also interested in investigating how different age groups interact with the VR tool, identifying any unique challenges or preferences that may arise and ensuring that the tool is accessible and effective for users of all ages and abilities.

### Limitations

The findings are constrained by the self-selection of patients interested in new technologies and a reliance on purposive sampling given the need to meet in person, both of which may not have resulted in a sample representative of the broader patient population. Consequently, the usability and acceptability of the technology might be overestimated as individuals with varied levels of technology savviness could encounter more difficulties. Future research should include a more diverse range of participants to ensure that the technology is accessible and effective for all users, particularly those from various care settings, rural areas, and non–English-speaking backgrounds. There may be potential biases as members of the research team also participated in development; however, this was important for the translation of the findings to the design of the prototype. In addition, this work did not explore whether the participants were executing the exercise correctly, which is planned as part of future enhancements. Future stages of the prototype should incorporate methods to evaluate the exercise intervention in the user’s preferred setting (eg, at home), include perspectives from physical therapists, and measure patient outcomes compared to current PT methods.

### Conclusions

In this study, we designed and evaluated the usability of a prototype for a VR-based PT intervention. While there was enthusiasm and interest in using a VR tool at home, participants were dissatisfied with the prototype’s usability, particularly related to the device itself. Usability issues were connected to the need for more instructions and difficulties interacting with the VR device. The findings highlight the importance of understanding users’ baseline knowledge of VR and creating effective onboarding materials. Future research should focus on extensive prototype testing and use multiple methods to evaluate the usability and acceptability of VR technology, as well as include an orientation to VR, as it was many participants’ first experience with the technology. Overall, VR tools have the potential to expand care access to rural and underserved communities and increase patient autonomy, making health care more engaging.

## Abbreviations

ADAPT XR: Advancing Digital Access to Physical Therapy via Virtual and Extended Reality Technology
PT: physical therapy
REDCap: Research Electronic Data Capture
SUS: System Usability Scale
VR: virtual reality
